# Underwater Acoustic Time Delay Estimation Based on Envelope Differences of Correlation Functions

**DOI:** 10.3390/s19051259

**Published:** 2019-03-12

**Authors:** Guodong Li, Jinsong Wu, Taolin Tang, Zhixin Chen, Jun Chen, Huang Liu

**Affiliations:** 1 Fishery Machinery and Instrument Research Institute, Chinese Academy of Fishery Sciences, Shanghai 200092, China; tangtaolin@fmiri.ac.cn (T.T.); chenzhixin@fmiri.ac.cn (Z.C.); chenjun@fmiri.ac.cn (J.C.); liuhuang@fmiri.ac.cn (H.L.); 2 Joint Laboratory for Deep Blue Fishery Engineering, Qingdao National Laboratory for Marine Science and Technology, Qingdao 266000, China; 3 Department of Electrical Engineering, Universidad de Chile, 8370451 Santiago, Chile

**Keywords:** time delay estimation, envelope differences of correlation functions, correlation method, underwater acoustic localization

## Abstract

This paper proposes underwater acoustic time delay estimation based on the envelope differences of correlation functions (EDCF), which mitigates the delay estimation errors introduced by the amplitude fluctuations of the correlation function envelopes in the traditional correlation methods (CM). The performance of the proposed delay estimation method under different time values was analyzed, and the optimal difference time values are given. To overcome the influences of digital signal sampling intervals on time delay estimation, a digital time delay estimation approach with low complexity and high accuracy is proposed. The performance of the proposed time delay estimation was analyzed in underwater multipath channels. Finally, the accuracy of the delay estimation using this proposed method was demonstrated by experiments.

## 1. Introduction

Time delay estimation has been a key issue in underwater acoustic localization, detection, and communications [1,2]. An early developed method for time delay estimation is the correlation algorithm, which estimates the time delay based on the peak of the correlation function [3,4]. The correlation algorithm and its improved algorithms, such as generalized correlation method [5], adaptive time delay estimation [6], generalized phase delay estimation [7], least mean square (LMS) time delay estimation [8,9], generalized phase spectrum algorithms [10], and so on, have been widely used due to their low computational complexities and high accuracies. In recent years, time delay estimation algorithms using broadband signals have also been extensively studied, and achieved good performance [11,12,13], However, in actual underwater acoustic systems, the received signals are affected by multipath fading channels and noise, especially when the noise is not ideal Gaussian white noise, and the signal sampling period is not infinitely long, and the traditional correlation delay estimation methods are used. In this case, it is difficult to accurately find the peak due to the amplitude fluctuations of the correlation function envelopes, which make the estimation accuracies of the delays greatly reduced [14,15].

The achieved accuracy of the time delay estimation via looking for the peak of the correlation function is limited by the sampling frequency. When the sampling frequency is low, the accuracy of time delay estimation is also low. To improve the resolution of the correlation peaks, it is usually necessary to interpolate the correlation peaks [16,17,18], using least squares method, cubic spline interpolation and other algorithms, which would not only increase the computation complexity, but also introduce new sources of errors. In underwater acoustic system, especially those involving underwater equipment, due to the limitation of equipment volume and power supply, it is impossible to use high complex computation method to estimate the time delay with high time delay estimation accuracy. Thus, we may need to find an algorithm that accurately estimates the time delay. In this paper, we propose an approach of the time delay estimation method based on envelope difference of correlation function to avoid the correlation peak detection, and thus avoid the influence of correlation peak amplitude fluctuation and improve the accuracy of time delay estimation. To solve the problem of sampling frequency limiting the time delay estimation accuracy, we propose a precise time delay estimation method with lower computational complexity.

## 2. Correlation Time Delay Estimation Method

The transmitted signal is: $x\left(0\right),x\left(T_{s}\right),\cdots ,x\left(kT_{s}\right),\cdots ,k=1,2,3,\cdots N$, where *N* is the number of consecutive samples, the sampling interval is $T_{s}$, and the measurement time is $N\times T_{s}$. Under the underwater acoustic multipath channel, the received signal can be assumed to be a linear superposition of multipath signals [19]. The discrete time model of the received signal is:

(1) $$\begin{array}{c} y\left(kT_{s}\right)=\sum_{d=1}^{D}a_{d}x\left(kT_{s}-\tau_{d}\right)+v\left(kT_{s}\right), \end{array}$$

where *D* is the number of multipath signals, $v(kT_{s})$ is additive Gaussian noise, $a_{d}$ is the amplitude fading of the *d*-path multipath signal, and $\tau_{d}$ is the arrival time delay, which is a real number greater than zero.

In active system, the known transmitted signal is used as the reference signal. The cross-correlation function of the reference signal $x(kT_{s})$ and the received signal $y(kT_{s})$ is given by

(2) $$\begin{array}{cc} R_{sx}\left(lT_{S}\right) & =E[x\left(kT_{S}\right)\;\;y^{*}\left(lT_{S}+kT_{S}\right)]\\ & =E\left[\sum_{d=1}^{D}x(kT_{s})a_{d}x^{*}\left(lT_{s}+kT_{S}-\tau_{d}\right)\right]+E\left[x\left(kT_{S}\right)v\left(lT_{s}+kT{}_{s}\right)\right]\\ & =\sum_{d=1}^{D}a_{d}R_{ss}\left(lT_{s}-\tau_{d}\right)+R_{sv}\left(lT_{s}\right), \end{array}$$

where * denotes complex conjugate.

If the additive noise is ideal uncorrelated white Gaussian noise, the reference signal and noise are uncorrelated. The cross-correlation of the reference signal and noise $R{}_{sv}\left(lT_{s}\right)$ is:

$$R{}_{sv}\left(lT_{s}\right)=0$$

Therefore, Equation (2) is expressed as

(3) $$\begin{array}{c} R_{sx}\left(lT_{s}\right)=\sum_{d=1}^{D}a_{d}R_{ss}\left(lT_{S}-\tau_{d}\right) \end{array}$$

From the characteristics of the autocorrelation function, we have

$$\left|R_{ss}\left(lT_{S}-\tau_{d}\right)\right|\approx \left|R(lT_{s}-Floor\left(\tau_{d}/T_{s}\right)T_{s})\right|\leq \left|R_{ss}\left(0\right)\right|,$$

where Floor() represents the floor function. When $lT_{s}=Floor\left(\tau_{d}/T_{s}\right)T_{s}$, $\left|R_{ss}\left(lT\right)\right|$ takes the maximum value, that is, the time corresponding to the peak of the correlation function is the time delay of the *d*th path.

## 3. Time Delay Estimation Method Based on Envelope Difference of Correlation Function

In the traditional correlation method, the additive noise is not ideal Gaussian white noise and the signal sampling period is not infinitely long in practical applications, so $R_{sv}\left(lT_{s}\right)$ may not necessarily strictly zero. When the signal-to-noise ratio is large or the noise is not correlated, the influence of $R_{sv}\left(lT_{s}\right)$ on the determination of the correlation peak maximum is negligible, that is, the correlation method can be used to obtain better time delay estimation performance. However, when the signal-to-noise ratio is low or the noise is correlated, the envelope of the correlation function severely fluctuates, so that $R_{sv}\left(lT_{s}\right)$ will affect the determination of the correlation peak maximum, which may reduce the accuracy of the time delay estimation by the correlation method.

**Figure 1:**
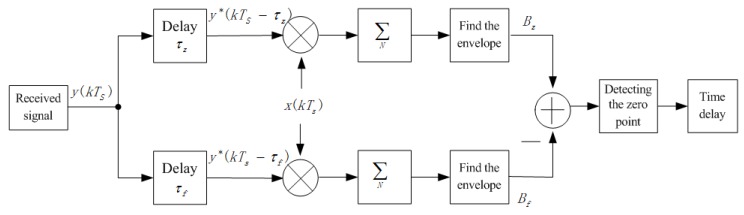
Principle of time delay estimation method based on envelope difference of correlation function.

As shown in Figure 1, we propose time delay estimation based on envelope difference of correlation function. The received signal $y(kT_{s})$ is correlated with the reference signal $x(kT_{s})$ after the delays $\tau_{z}$ and $\tau_{f}$, respectively,

$$\begin{array}{c} R_{s1x}\left(lT_{s}\right)=\sum_{d=1}^{D}a_{d}R_{ss}\left(lT_{s}-\tau_{d}-\tau_{z}\right)\\ R_{s2x}\left(lT_{s}\right)=\sum_{d=1}^{D}a_{d}R_{ss}\left(lT_{s}-\tau_{d}-\tau_{f}\right) \end{array},$$

where $\tau_{z}=l_{z}T_{s}$ and $\tau_{f}=l_{f}T_{s}$.

We assume that $R_{ss}\left(lT_{s}\right)$ is a bandpass signal near carrier frequency $f_{0}$, which can be expressed in a complex form

(4) $$\begin{array}{c} R_{ss}\left(lT_{s}\right)=A\left(lT_{s}\right)\exp j[2\pi f_{0}lT_{s}+\theta \left(lT_{s}\right)], \end{array}$$

where $A(lT_{s})$ and $\theta (lT_{s})$ are amplitude and phase modulation functions of $R_{ss}\left(lT_{s}\right)$ relative to carrier frequency *B*, respectively, $R_{ss}\left(lT_{s}\right)$ can be expressed as

(5) $$\begin{array}{c} R_{ss}\left(lT_{s}\right)=RC_{ss}\left(lT_{s}\right)e^{j2\pi f_{0}lT_{S}}, \end{array}$$

and $RC_{ss}\left(lT_{s}\right)=A\left(lT_{s}\right)e^{j\theta (lT_{s})}$ is called complex envelope.

The correlation function follows the relation

$$R_{ss}\left(-lT_{s}\right)={R_{ss}}^{*}\left(lT_{s}\right).$$

Note that

$$\begin{array}{c} RC\left(-lT_{s}\right)e^{\;-\;j2\pi f_{0}lT_{s}}=R{C^{*}}_{ss}\left(lT_{s}\right)e^{-j2\pi f_{0}lT_{S}},\\ RC\left(-lT_{s}\right)=R{C^{*}}_{ss}\left(lT_{s}\right). \end{array}$$

Thus,

(6) $$\begin{array}{c} B_{ss}\left(lT_{s}\right)=\left|RC_{ss}\left(lT_{s}\right)\right|=\left|R{C^{*}}_{ss}\left(lT_{s}\right)\right|=B_{ss}\left(-lT_{s}\right), \end{array}$$

where the real envelope $B_{ss}\left(lT_{s}\right)$ of function $R_{ss}\left(lT_{s}\right)$ is an even function.

In active system, when d=1, the time delay estimation of the 1th path is particularly important for the system applications. If $B_{ss1z}\left(lT_{s}-\tau_{1}-\tau_{z}\right)$ and $B_{ss1f}\left(lT_{s}-\tau_{1}-\tau_{f}\right)$ are the envelopes of $R_{ss}\left(lT_{s}-\tau_{1}-\tau_{z}\right)$ and $R_{ss}\left(lT_{s}-\tau_{1}-\tau_{f}\right)$, that is, $B_{ss1z}\left(lT_{s}-\tau_{1}-\tau_{z}\right)$ and $B_{ss1f}\left(lT_{s}-\tau_{1}-\tau_{f}\right)$ are even functions, and are symmetric about $\tau_{1}+\tau_{z}$ and $\tau_{1}+\tau_{f}$, respectively, which is:

$$B_{ss1z}\left(lT_{s}\right)=B_{ss1f}\left(lT_{s}+\tau_{f}-\tau_{z}\right)$$

We could calculate the envelope difference as

$$DB\left(lT_{s}\right)=B_{ss1z}\left(lT_{S}\right)-B_{ss1f}\left(lT_{s}\right)=B_{ss1f}\left(lT_{s}+\tau_{f}-\tau_{z}\right)-B_{ss1f}\left(lT_{s}\right),$$

when

$$lT_{s}=\tau_{1}+\tau_{f}+\frac{\tau_{z}-\tau_{f}}{2}.$$

Thus,

(7) $$\begin{array}{cc} DB(\tau_{1}+\tau_{f}+\frac{\tau_{z}-\tau_{f}}{2}) & =DB(\tau_{1}+\frac{\tau_{z}+\tau_{f}}{2})\\ & =B_{ss1f}\left(\tau_{1}+\tau_{f}-\frac{\tau_{z}-\tau_{f}}{2}\right)-B_{ss1f}\left(\tau_{1}+\tau_{f}+\frac{\tau_{z}-\tau_{f}}{2}\right). \end{array}$$

Since $B_{ss1z}\left(\tau -\tau_{1}-\tau_{z}\right)$ and $B_{ss1f}\left(\tau -\tau_{1}-\tau_{f}\right)$ are even functions, and are symmetric about $\tau_{1}+\tau_{z}$ and $\tau_{1}+\tau_{f}$, respectively, we know that:

$$DB(\tau_{1}+\frac{\tau_{z}+\tau_{f}}{2})=0$$

If $\tau_{z}=-\tau_{f}$, there are:

$$DB\left(Floor\left(\tau_{1}/T_{s}\right)T_{s}\right)\approx DB\left(\tau_{1}\right)=0$$

This proposed approach can estimate the time delay via detecting the zero point of envelope difference function, and avoid detecting the peak of the correlation function to estimate the time delay.

## 4. Differential Delay Determination

For the time delay estimation based on envelope differences of correlation functions, the selection of the differential delay $\tau_{z}$ is crucial for the achieved accuracy of the time delay estimation, and the appropriate delays can also reduce the interferences of the multipath channels. We determined the selection range of the differential delay $\tau_{z}$ via simulation analysis.

**Figure 2:**
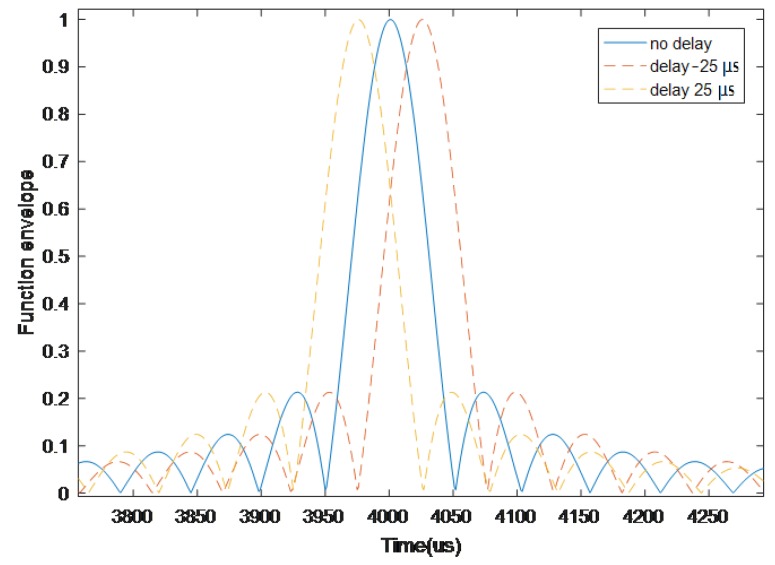
The correlation function envelope.

Simulation settings included the chirp frequency modulation (FM) signal with frequency-band from 60 kHz to 80 kHz and the signal time period 4 ms. It can be known from the characteristics of the correlation function that the main peak width of the correlation function envelope is proportional to the inverse of the signal bandwidth, that is, the main peak width *K* of the correlation function envelope is about twice the inverse of the effective signal bandwidth. As shown in Figure 2, *K* was equal to 100 μs.

**Figure 3:**
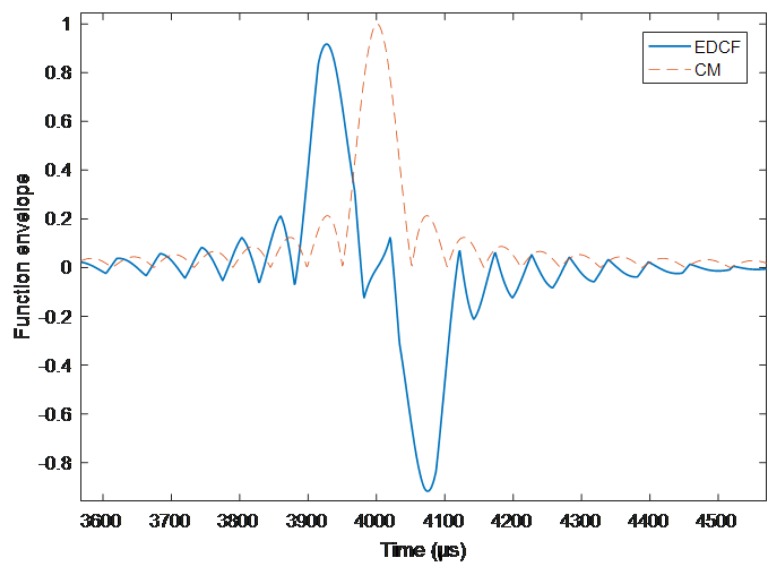
Differential correlation results when $\tau_{z}=70\;\mu$s.

If the differential delay $\tau_{z}$ is greater than the width *K*/2, the results of the differences will appear in the case with more fluctuation points near the zero value, which will affect the determination of the time delay, as shown in Figure 3.

**Figure 4:**
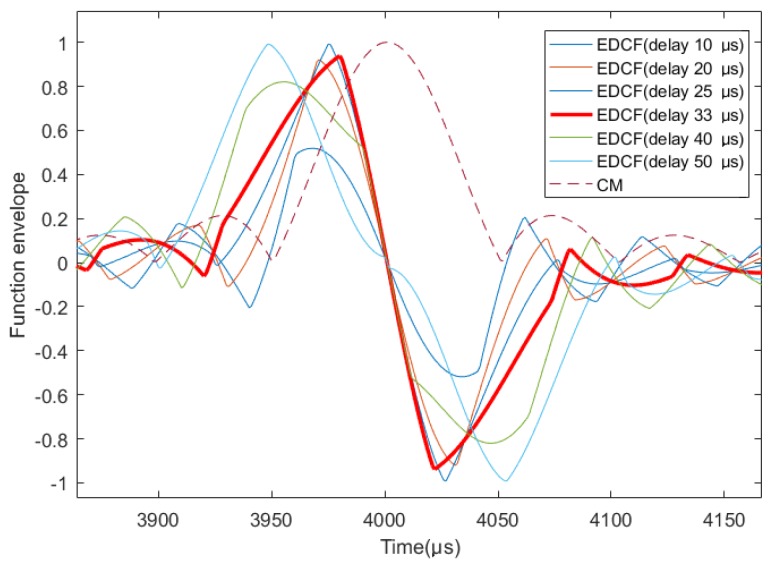
Differential correlation results when $\tau_{z}\leq 50\;\mu$s.

Figure 4 shows the various cases when the differential delay $\tau_{z}$ is less than the width *K*/2. It can be seen that, when the differential delay $\tau_{z}$ is equal to *K*/3, the correlation envelope difference function has the largest slope when crossing the zero point (in the case of a thick red line in the figure). Since the slope of the zero-crossing point is small and susceptible to fluctuations due to interferences, the occurrence of repeated zero-crossing is not conducive to the delay estimation, and thus differential delay $\tau_{z}$ is chosen as *K*/3.

## 5. Precise Time Delay Calculation

If the time delay estimation based on correlation envelope differences is performed via digital samplings, zero-crossing points cannot be directly solved due to the sampling intervals. Therefore, the accuracy of the time delay estimation is less than $T_{s}$ in the traditional correlation methods, and thus the solution usually is to interpolate the correlation peaks to adjust the correlation peaks, which may greatly increase the signal processing computational complexity.

In the proposed correlation envelope difference based approach, the exact delay estimation point is not at the top of the correlation peak but at the maximum position of the slope differences of function envelopes. Based on this observation, we propose a two-point linear approximation method for accurate time delay estimation.

**Figure 5:**
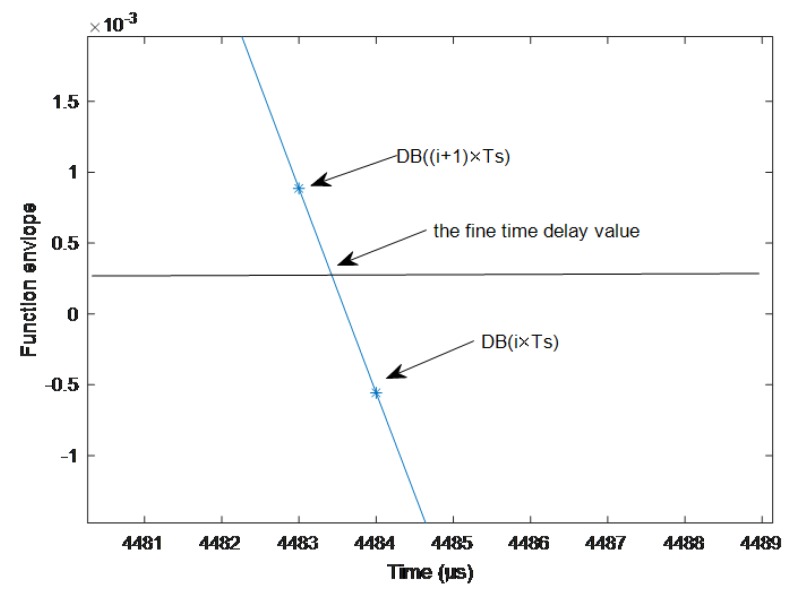
Schematic diagram of two-point linear approximate exact solution time delay.

As shown in Figure 5, the difference of correlation function envelopes follows

$$\begin{array}{c} DB(iT_{s})\leq 0\\ DB(iT_{s}+T_{S})>0 \end{array}$$

Then, the fine time delay value is calculated as

(8) $$\begin{array}{c} \tau_{b}=\frac{DB(iT_{s})}{DB\left(iT_{s}+T_{s}\right)-DB\left(iT_{s}\right)}\times T_{s}+i\times T_{s} \end{array}$$

This proposed approach can overcome the conditions of limited samplings rate without increasing computation complexity due to the use of complex interpolation algorithms.

## 6. Anti-Multichannel Channel Performance Analysis

**Figure 6:**
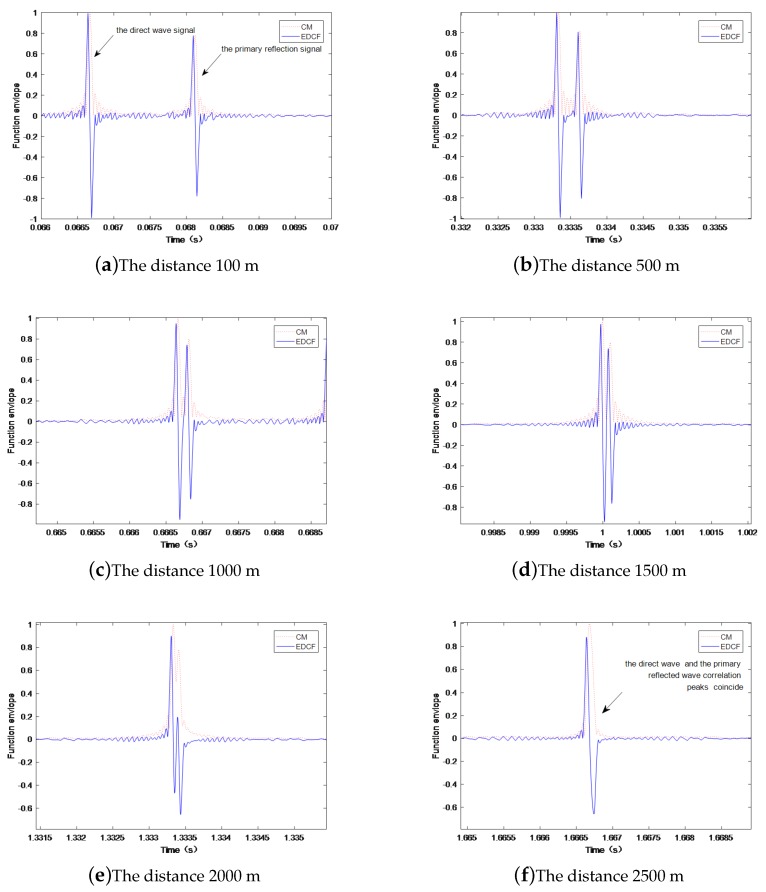
The anti-multipath channel performance of the method.

In shallow sea conditions, multipath channels have significant impacts on time delay estimation. In Equation (1), $d_{1}$ is the direct wave signal, and $d_{2}$ is the primary reflection signal on the seabed or the sea surface, and the main reason for the good accuracy in the time delay estimation is the interference of the primary reflected signal $d_{2}$ in the shallow sea. The envelopes differences of correlation functions at different working distances under multipath channels were investigated via the simulations presented below. Simulation conditions: The sea water depth was 50 m, the receiving transducer and the transmitting transducer were laid at a depth of 10 m, the sound velocity was 1500 m/s. The performance under different multipath channels was analyzed via changing the distance between the receiving transducer and the transmitting transducer, as shown in Figure 6.

**Figure 7:**
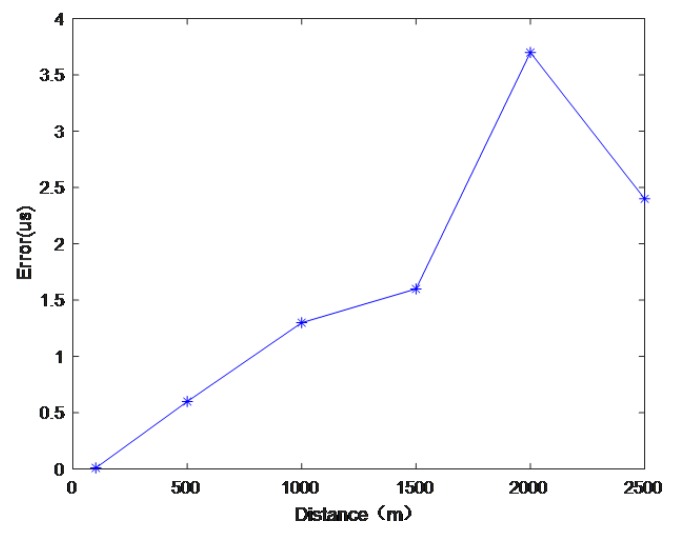
Shows the error of multipath delay calculation at different distances.

The simulation results show that, when the distance between the transmitter and the transmitter was less than 1000 m, the time delay solution was basically not affected by multipath interferences. When the distance was between 1000 m and 2000 m, the influence of multipath interferences became larger. However, when the distances were greater than 2000 m, since the direct wave signal and the primary reflected wave correlation peaks substantially coincided, the influences of multipath interferences on the time delay solution gradually reduced. Figure 7 shows the errors of multipath time delay estimation at different chosen distances.

## 7. Experimental Data Analysis

The performance of the time delay estimation algorithm proposed in this paper was evaluated through sea trials. The experiments were conducted in the East China Sea near Zhongkuai Island. The water depth was 50 m, the distance of transmitting and receiving was about 100 m, the laying depth of transmitting and receiving transducer was 10 m, the transmitter power was 20 watts, and the received signal-to-noise ratio was about 15 dB. The transmitting signal was from 60 kHz to 80 kHz chirp FM signal and signal time period was 4 ms.

**Figure 8:**
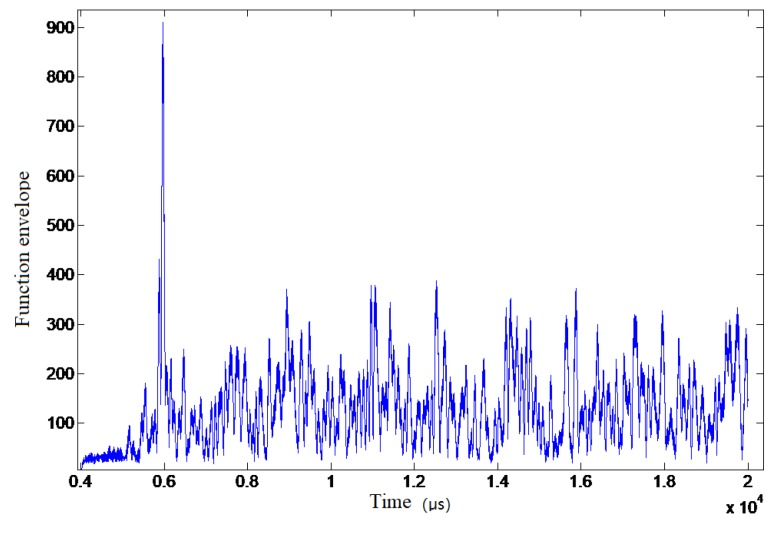
The correlation function envelope in test data.

The correlation function envelope in experimental data (for ease of analysis, the data were truncated, and all figures below) is shown in Figure 8.

**Figure 9:**
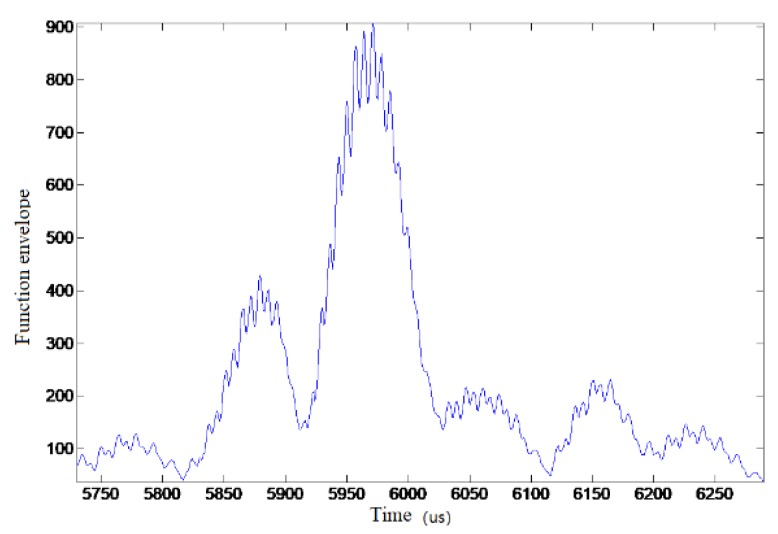
The correlation function envelope in test data (the main peak).

Figure 9 is an enlarged view of the main peaks of the envelope. It can be seen that the envelopes of the main peaks fluctuated extremely and it was difficult to accurately obtain the maximum values to estimate the correct time delay values, which is the main reason for the low achieved accuracy using the traditional correlation time delay estimation.

**Figure 10:**
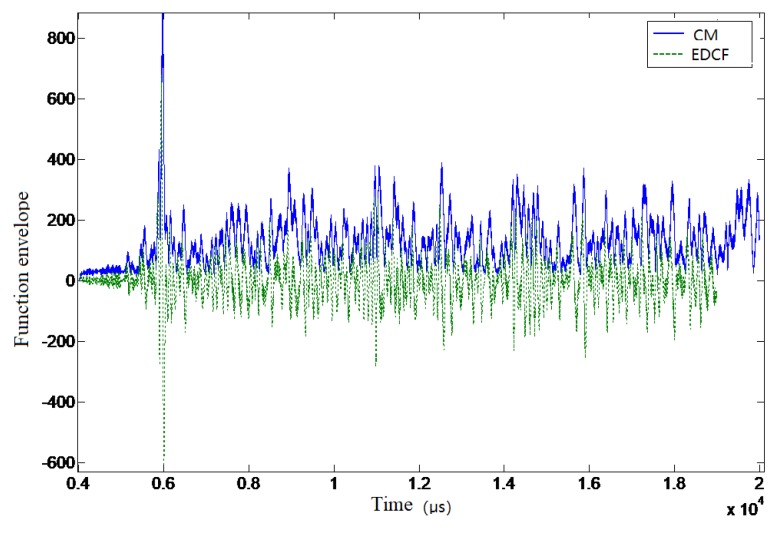
The correlation envelope function difference result in test data.

**Figure 11:**
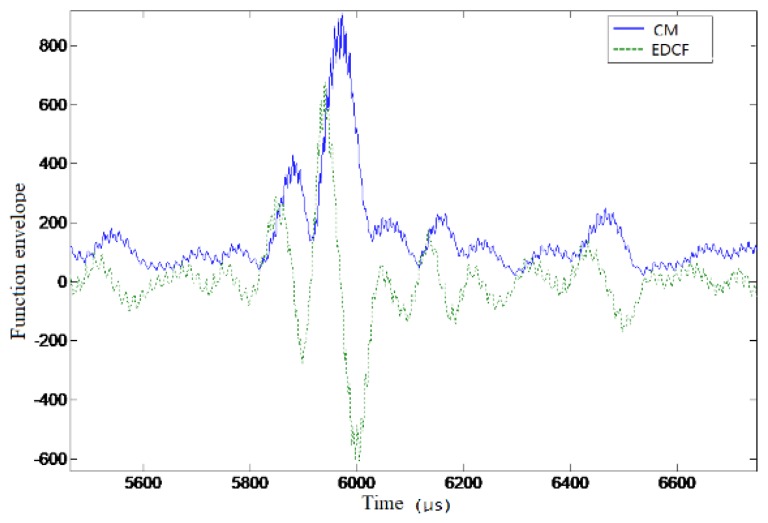
The correlation envelope function difference result in test data (the main peak).

Figure 10 and Figure 11 show the results of the time delay estimation based on the differences of correlation envelopes. It can be seen that there was no large amplitude fluctuation in the zero-crossing points of the curves, which could effectively improve the accuracy of the time delay estimation.

Table 1 shows the time delay estimation results of fixed-point measurement test data using the correlation method and the correlation envelope function difference method. The mean of time delay using the correlation method was 5806.4 μs with standard deviation of 4.10 μs, and the mean of time delay using the differences of correlation envelopes was 5807.2 μs with standard deviation of 0.25 μs. The experimental results show that the approach based on the differences of correlation function envelopes significantly improved the accuracies of time delay estimation.

## 8. Conclusions

In this paper, an approach based on the differences of correlation function envelopes has been proposed to estimate the time delay, which can avoid the delay estimation errors caused by the peak amplitude fluctuations of the correlation function envelope and significantly improve the accuracy of the time delay estimation. Under the condition of underwater acoustic multipath channels, the proposed method exhibits excellent performances under different multipath interferences. For time delay estimation in digital sampling systems, the proposed method can overcome the limited sampling frequencies without increasing computation complexities. 

## Figures and Tables

**Table 1:** Comparison of time delay estimation performance between the correlation method and the correlation function envelope difference method.

Number	Result in CM (μs)	Result in EDCF (μs)	Number	Result in CM (μs)	Result in EDCF (μs)
1	5801	5807.3	11	5808	5807.1
2	5808	5807.7	12	5807	5807
3	5806	5807	13	5800	5807.2
4	5808	2807.7	14	5807	5807.1
5	5800	5807.8	15	5807	5807
6	5807	5807.1	16	5800	5807.2
7	5805	5807	17	5818	5807
8	5811	5807.1	18	5807	5807
9	5807	5807.1	19	5807	5807.1
10	5806	5807.2	20	5807	5807
